# Pulmonary arterial hypertension before closure of a persistent arterial duct (PDA) in children <12 months: clinical implications and outcome

**DOI:** 10.1016/j.ijcchd.2026.100718

**Published:** 2026-10-08

**Authors:** Alessia Callegari, Mathilde Meot, Damien Bonnet, Sophie Malekzadeh-Milani

**Affiliations:** a M3C-Necker, Hôpital Necker-Enfants Malades, AP-HP, Université de Paris Cité, Cardiologie Congénitale et Pédiatrique, Paris, France; b University Children's Hospital of Zurich, Departement of Pediatric Cardiology, Zurich, Switzerland; c Université de Paris Cité, Paris, France

**Keywords:** PDA closure, Pulmonary hypertension, Neonate, Heart catheterization, Pulmonary pressures

## Abstract

**Background:**

The management of patent ductus arteriosus (PDA) in infants with pulmonary hypertension (PH) remains controversial due to concerns regarding irreversible pulmonary vascular disease and post-closure hemodynamic deterioration. Evidence in children <12 months is limited. We aim to describe the hemodynamics and outcomes of infants with isolated PDA and PH undergoing invasive evaluation and to identify clinical factors associated with PH persistence after closure.

**Methods:**

We retrospectively analyzed infants <12-months with isolated PDA and mean pulmonary artery pressure (mPAP) > 20 mmHg who underwent right heart catheterization. Hemodynamics, procedural strategy, and clinical outcomes were assessed at 6-/12-months, and last follow-up.

**Results:**

A total of 220 patients were included (mean age 154 ± 87 days; weight 5.7 ± 1.7 kg). mPAP was 36.5 ± 12.8 mmHg and Qp/Qs 1.9 ± 1.0. PDA closure was performed in 214 patients (186 transcatheter, 28 surgical). Cumulative mortality was 1.8%, occurring exclusively in patients with severe comorbidities. Persistent PH was noted in 8 patients (3.6%) at 6-months and 2 patients (0.9%) at 12-months and long-term follow-up. No cases of PH relapse were observed after successful closure. All persistent PH cases were associated with developmental lung disease or severe syndromic conditions. Infants without major comorbidity demonstrated resolution of PH after closure.

**Conclusions:**

In infants with isolated PDA and PH, ductal closure within the 1 year is associated with near-universal normalization of pulmonary pressures. Persistent PH is uncommon and primarily confined to patients with developmental lung disease. Clinical and echocardiographic assessment combined with invasive hemodynamic should guide operability decisions.

## 1 Introduction

Patent ductus arteriosus (PDA) is one of the most common congenital heart defects (CHD). In these cases, the persistence of arterial duct can result in significant left-to-right shunting and pulmonary overcirculation, leading to hemodynamic compromise [1,2]. While many PDAs close spontaneously in term neonates, persistent patency beyond the neonatal period, especially in preterm infants or those with lung developmental disease (LDD), can promote progressive pulmonary vascular remodeling and ultimately the development of pulmonary hypertension (PH) [[3], [4], [5]].

According to the 6th World Symposium on Pulmonary Hypertension, PH is defined as a mean pulmonary arterial pressure (mPAP) > 20 mmHg, measured via right heart catheterization (RHC) at sea level in both adults and children older than 3 months [[6], [7], [8]]. In the context of PDA, chronic exposure of the pulmonary circulation to increased flow and pressure contributes to intimal proliferation, vascular remodeling, and smooth muscle hypertrophy, eventually elevating pulmonary vascular resistance (PVR) [4,9]. These changes may occur early, particularly in vulnerable infants with very low birth weight, bronchopulmonary dysplasia (BPD), or underlying LDD [2,5,10]. Historically, concerns regarding procedural risk, particularly the potential for persistent or worsened PH and right ventricular failure, led to cautious consideration of PDA closure in patients with established PH [1,11]. Moreover, in patients with advanced pulmonary vascular disease, the development of Eisenmenger physiology may function as a pressure-relief mechanism for the right ventricle. Nowadays, advances in transcatheter devices and interventional techniques have improved procedural safety, enabling technically successful closure even in very small infants, thereby limiting prolonged exposure to pulmonary overcirculation and PH [9,12]. A “treat-and-repair” or “repair-and-treat” strategy has also proven feasible in selected cases [9].

Despite increasing data on PDA closure in older children and adults with PH, evidence in infants under one year remains limited. In particular, little is known about how closure affects long-term PH outcomes in subgroups with LDD, chromosomal syndromes, or elevated PVR in early life. Therefore, this study aims to evaluate the clinical and hemodynamic profiles of infants under 12 months of age with isolated PDA who underwent invasive assessment, and to assess their long-term outcomes, identifying predictors of PH persistence and subgroups most likely to benefit from intervention.

## 2 Methods

### 2.1 Patient inclusion

From 2000 to 2024, we retrospectively analyzed data from all patients < 12 months of age with CHD consistent with an isolated PDA who had an invasive haemodynamic evaluation with RHC and weighted >2 kg. A total of 305 patients met these including criteria and only the 220 patients with PH defined as mPAP > 20 mmHg were included, 85 with mPAP < 20 mmHg were excluded. All parents signed an informed consent, and the study was approved by our institution ethics committee (MR004 n°20220729172242).

### 2.2 Hemodynamic parameters

The cardiac output (CO) was measured using the Fick method with assumed oxygen consumption (VO_2_) based on the LaFarge and Miettinen formula, recognizing that this method may introduce bias. Pulmonary and systemic blood flows, including Qp/Qs, were derived from oxygen content differences. Pulmonary vascular resistance (PVR) was calculated as the transpulmonary pressure gradient, defined as mean pulmonary artery pressure minus pulmonary arterial wedge pressure or left atrial pressure when available, divided by pulmonary blood flow (Qp). Indexed pulmonary vascular resistance (PVRi) was calculated using indexed pulmonary blood flow (Qpi) equivalent to PVR × BSA, and expressed in Wood units·m^2^. Serial blood gas analyses were obtained to confirm adequate pulmonary vasodilation and to account for dissolved oxygen, including measurement of PaO_2_, particularly during testing under increased inspired oxygen concentrations. A positive acute vasodilator test (AVT) was defined as a ≥20% reduction in PVRi and PVR/SVR ratio, with final values < 6 Wood units·m^2^ and <0.3, respectively, accompanied by an increase in Qp/Qs during vasodilator testing [11], [12], [13]. For Qp/Qs calculation, when feasible, we directly sampled pulmonary venous (PV) saturation from both left and right pulmonary veins. If this was not technically possible and systemic arterial saturation was normal, we assumed PV saturation to be near 98–100%. In cases with cyanosis and without PV access, estimations were made on a case-by-case basis, incorporating clinical context and diagnosis. In infants <3 months, for whom no separate universally accepted invasive haemodynamic threshold is available, mPAP >20 mmHg was used as an operational study definition.

Mode of anaesthesia remained the same across patients, and was performed as previously described [14].

Operability was assessed individually using integrated clinical, echocardiographic, and haemodynamic findings rather than predefined pulmonary pressure or PVRi thresholds. Continuous right-to-left ductal shunting and severe comorbidity were considered important factors against immediate closure.

Formal balloon or temporary device occlusion testing was not routinely performed; in selected borderline cases, haemodynamic stability was observed with the device in position before final release, and no procedure required abortion because of haemodynamic deterioration.

For outcome data, patients were evaluated during routine outpatient cardiology follow-up at approximately 6 months, 12 months, and at the last available follow-up. PH persistence or resolution was assessed primarily by transthoracic echocardiography in conjunction with clinical evaluation. Repeat cardiac catheterization was not routinely performed. PH was considered persistent when echocardiography continued to show evidence of elevated pulmonary arterial pressure and resolved when pulmonary pressure assessment normalized.

### 2.3 Statistical analysis

Statistical analysis was performed using SPSS 29 (SPSS Inc, IBM Company, Chicago Illinois). Continuous variables are expressed as mean (SD), and categorical data as counts and percentages.

## 3 Results

### 3.1 Population description

A total of 220 patients were included. Mean age was 154.4 days (SD: 87 days), 64.1% of the patients were female. Patients had a mean weight of 5.7 kg (SD: 1.7 kg). The mean height was 62.7 cm (SD: 7.5 cm).

### 3.2 Diagnosis and indication for RHC

Primary reason for cardiac catheterization in this cohort was closure of PDA, accounting for 94.1% (n = 207) of the cases. Other reasons included suspected PH based on echocardiogram findings in isolated PDA (2.7%, n = 6), and shunt calculation (3.2%, n = 7).

Table 1 provides a detailed overview of the clinical diagnoses found at heart catheterization. Pulmonary diseases were present in 17.3% of the patients, with severe bronchopulmonary dysplasia (BPD) accounting for 4.5% of cases. In terms of syndromic diseases, chromosomal anomalies were identified in 18.6% of the patients, with T21 (11.8% of the cohort) also being a major contributor to this group. Other syndromes, including Pierre Robin Sequence, Di George Syndrome, CHARGE Syndrome, and Sotos Syndrome, were present in smaller proportions, ranging from 0.5% to 1.8%.

**Table 1:** Patients’ comorbidities.

	n	%
**Developmental Lung Disease**	38	17.30%
Alveolocapillary Dysplasia	1	0.50%
CDH	1	0.50%
Severe BPD	10	4.50%
Trisomy 21 (T21)	26	11.80%
**Syndromal Diseases**		
Chromosomal Anomaly	41	18.60%
T21	26	11.80%
Pierre Robin Sequence	1	0.50%
Di George Syndrome	1	0.50%
CHARGE Syndrome	4	1.80%
Sotos Syndrome	2	0.90%

### 3.3 Procedural outcome and hemodynamic findings

The majority of the patients (89.5%) underwent both left and right catheterization. PVRi calculation was available in 86 (39%) patients. Pulmonary hypertension testing using AVT was conducted in 10 (4.5%) patients. Out of those, 2 patients were found to be non-vasoreactive. The mean procedural time for cardiac catheterization was 38.6 min (SD: 27.3). There were no major complications reported during the procedures. There were two device migration/embolization events; both devices were retrieved, and both patients subsequently underwent surgical ligation. Other uncommon procedural events included a total of 5 clinically not relevant device-related aortic or pulmonary arterial obstruction.

The mean pulmonary artery pressure (mPAP) was 36.5 mmHg (SD = 12.8 mmHg). Mean aortic pressure (mAoP) was 62.5 mmHg (SD = 12.5 mmHg), systolic 85.5 mmHg (SD = 15.2 mmHg) and diastolic 42.3 mmHg (SD = 11.4 mmHg) with a widened pulse pressure (43.2 mmHg, SD = 12.6 mmHg), consistent with significant ductal runoff physiology. mPAP to mAo ratio was >1 in 1.4%, 1 in 19.5% and <1 in 79.1%. The ratio of mean pulmonary artery pressure to mean aortic pressure (mPAP/mAoP) was 0.6 (SD = 0.2). The mean Qp/Qs ratio, representing pulmonary-to-systemic flow, was 1.9 (SD = 1.0). Indexed pulmonary vascular resistance (PVRi) (n = 86) was 3.0 Wood Units (WU) (SD = 2.1 WU. The PVRi/iSVR ratio has a mean of 0.5 (SD = 0.4). Haemodynamic results are showed in Table 2. In the subgroup of patients at risk (n = 32), defined with mPAP ≥50 mmHg, mean mPAP was 60.3 mmHg (range 50–84), with systemic or suprasystemic pulmonary pressures in 23 patients. PVRi was available in 7 patients and ranged from 2.97 to 9.44 WU·m^2^.

**Table 2:** Hemodynamics.

Hemodynamic	Mean	SD
**SAoO2 (%)**	93.2	8.5
MVO2 (%)	65.9	13.7
RAmP (mmHg)	7	2.7
PVO2 (%)	98	3.4
PAO2 (mmHg)	83.7	7.8
Qp:Qs	1.9	1
sPAP (mmHg)	50	17
dPAP (mmHg)	24.4	10.2
mPAP (mmHg)	36.5	12.8
PAWP (mmHg)	9.7	3.6
aPVRi (WU^a^m2)	3	2.1
aSVRi(WU^a^m2)	8.7	8.2
aPVRi/SVRi	0.5	0.4
sAoP (mmHg)	85.5	15.2
mAoP (mmHg)	62.5	12.5
dAoP (mmHg)	42.3	11.4
mPAP/mAO	0.6	0.2
Pulse pressure	43.2	12.6

**Severity of PH**	**n**	**%**

mPAP < mAoP	174	79.10%
mPAP = mAoP	43	19.50%
mPAP < mAo	3	1.40%

a Available for 86 patients.

PDA diameter on angiography was 5.3 (SD = 2.0 mm (range 2.3–7.4 mm)). Closure of PDA through catheterization was performed in 186 patients, representing 84.5% of patients. Furthermore, 28 (12.7%) underwent surgical PDA ligation, with 18 of these procedures occurring when an interventional approach was not possible.

Detailed procedural information was available for 144 of 186 transcatheter PDA closures (66.7%). Among procedures with documented device type, ADO/Amplatzer-type duct occluders, including ADO I and ADO II/II AS, were the most frequently used devices (89.4%), followed by coils (18.2%), AVP devices (6.1%), and KONAR/MFO devices (3.0%).

### 3.4 Outcome

Detailed patient outcomes are shown in Table 3 and Fig. 1.

**Table 3:** Outcome.

Outcome	6 months		12 months		Long-term	
	n	%	n	%	n	%
**Cause of death**	2	0.9%	2	0.9%	0	0.0%
- Cardiac failure	0	0.0%	0	0.0%	0	0.0%
- Postoperative complications	0	0.0%	0	0.0%	0	0.0%
- Polymorbidity or palliative care without PDA closure	2	0.9%	0	0.0%	0	0.0%
- PH crisis	0	0.0%	0	0.0%	0	0.0%
- Respiratory failure	0	0.0%	2^a^	0.9%	0	0.0%
**Persistent PH**	8	3.6%	2^b^	0.9%	2^b^	0.9%
**Relapse of PH after shunt closure**	0	0.0%	0	0.0%	0	0.0%
**PH therapy**	8	3.6%	2	0.9%	2	0.9%
- Bosentan	0	0.0%	1	0.5%	1	0.5%
- Sildenafil	4	1.8%	0	0.0%	0	0.0%
- Bosentan + Sildenafil	1	0.5%	1	0.5%	1	0.5%
- + Prostacycline	0	0.0%	0	0.0%	0	0.0%
- O2 therapy	3	1.4%	0	0.0%	0	0.0%

a No PDA closure in 1, PDA closure in 1.

b Alveolocapillary dysplasia (no PDA closure) and severe BPD (PDA closure at 10-months after treat&repair strategy), same patients.

**Fig. 1:**
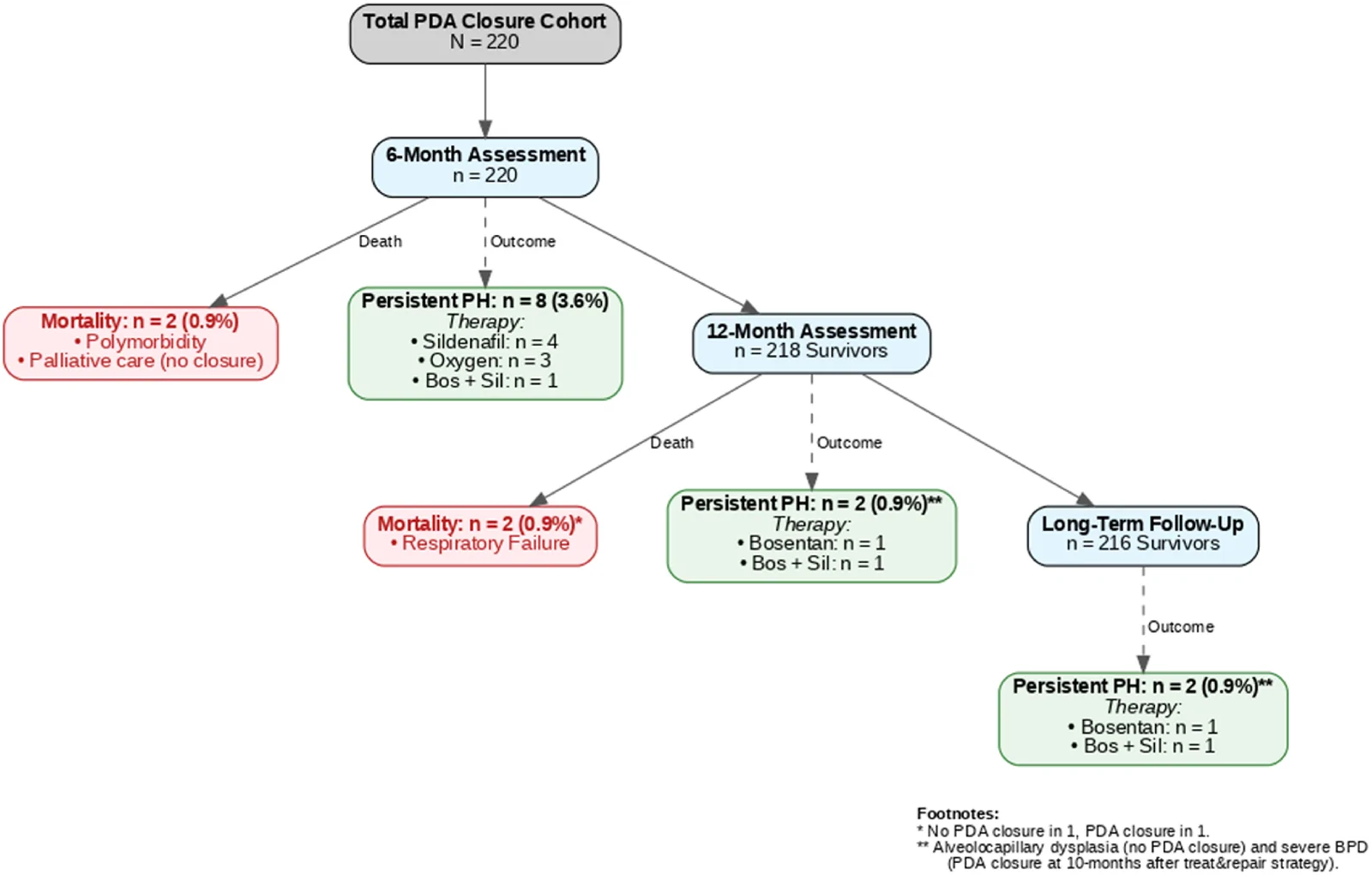
Longitudinal patient outcomes Post-PDA closure.

#### 3.4.1 Outcome at 6-months

A total of 2 deaths were recorded, representing 0.9% of the cohort, both in patients who did not undergo PDA closure due to polymorbidity and decision for palliative care. PH persisted in 8 patients (3.6%), all with LDD, 2 of which with genetic syndrome (T21 and CHARGE syndrome). Individual clinical characteristics, baseline haemodynamics, PDA management, PH therapy, and subsequent outcomes of these patients are summarized in Supplementary Table S1. Pulmonary hypertension therapy was given to all 8 patients, sildenafil was used in 4 patients (1.8%), while 1 patient (0.5%) received a combination of bosentan and sildenafil. Three patients (1.4%) required oxygen therapy. No cases of PH relapse after PDA closure and primary resolution of PH were reported.

#### 3.4.2 Outcome at 12-months

At 12-month follow-up, 2 further patients died, with one death occurring after refusal for PDA closure and another after PDA closure. Respiratory failure in the context of LDD was reported in both patients. PH persisted in 2 of the previously described patients, and no cases of PH relapse after PDA closure and PH resolution were recorded. One patient had alveolocapillary dysplasia and no PDA closure was performed. The second patient had severe BPD and PH persistence after surgical PDA closure with a treat&repair strategy at 10 months of age. PH therapy was administered to both patients. Sildenafil was used in the first patient, and a combination of bosentan and sildenafil in the other. Of the other patients who had PH at 6 months, 4 cases resolved, while the other 2 patients are those who died.

#### 3.4.3 Long term outcome

At long-term follow-up (mean follow-up time of 2.44 years (SD: 2.99 years)), no further patients died. PH was observed in the same 2 patients of the 12-months follow-up and both patients remained on PH therapy. No cases of PH relapse after PDA closure and primary PH resolution were observed.

#### 3.4.4 Patients without PDA closure

Immediate PDA closure was not performed in 6 patients. Within this group there were 3 deaths: 2 due to polymorbidity (LDD and LDD with Trisomy 21) and decision for palliative care, and 1 with LDD due to severe BPD and contraindication for PDA closure due to continuous right-to-left shunt. Among the 3 survivors, one had alveolocapillary dysplasia and clinical contraindication for PDA closure due to continuous right-to-left shunt, while 2 patients were treated & repaired: in 1 case of Trisomy 21 PH resolved at 12-months, while the second patients had no resolution of PH after shunt closure. Both these last patients had negative response at AVT.

## 4 Discussion

Our findings support the feasibility of PDA closure in presence of PH in infants diagnosed before one year of age, while also emphasizing the importance of patient selection and comorbidities.

In our cohort, no relapses of PH after PDA closure were observed in patients with isolated PDA and no syndromic or lung comorbidities. This is consistent with prior literature suggesting that PH driven primarily by volume and pressure overload from PDA remains potentially reversible with closure, although the present study did not perform an age-stratified analysis to confirm a specific benefit of earlier intervention [15,16]. In this selected cohort of infants with PDA-associated PH, operability was assessed using an integrated clinical, echocardiographic, and haemodynamic approach, and pulmonary pressure values alone were not used as absolute exclusion criteria. Infants demonstrating clear left-to-right shunting with evidence of pulmonary overcirculation and reduced systemic diastolic pressure were generally considered suitable candidates for closure. Importantly, this strategy relied on integrated pre-catheterization clinical and echocardiographic assessment rather than on isolated invasive parameters. The native duct diameter was consistent with hemodynamically significant shunts. Additionally, invasive aortic pressure measurements demonstrated relatively low diastolic pressures (42.3 ± 11.4 mmHg) and widened pulse pressure (43.2 ± 12.6 mmHg), supporting the presence of ductal runoff physiology in the majority of patients selected for closure.

Our results are consistent with other reports demonstrating favourable hemodynamic responses post-intervention [1,9,10]. Vijayalakshmi et al. observed that device closure in patients with severe PH led to normalization of pressures in most cases, especially when closure occurred at a younger age [10]. Similarly, Shah et al. reported sustained improvements in mean pulmonary artery pressures and functional status in children undergoing closure despite elevated baseline pressures [11].

These outcomes support the notion that PH in infancy, when primarily driven by volume and pressure overload from PDA, remains potentially reversible if addressed early [15,16]. Wu et al. emphasized that PDA size indexed to body surface area correlates significantly with mPAP, suggesting that larger shunts contribute more aggressively to vascular remodeling and should prompt earlier intervention [12]. Lammers et al. observed a small but notable risk of late-onset PH in patients with previously closed shunts, though this was more common in older patients and those with associated anomalies [4]. The affected patients had all comorbidities, namely LDD, in accordance to the literature [17]. In fact, infants with LDD, in our cohort, showed less favourable outcomes, indicating that these conditions are not transient and can lead to persistent PH. This is consistent with the findings of previous research on the long-term impact of LDD on PH outcomes, showing non-transient PH in 34-49% [7,15]. Philip et al. noted that prolonged ductal patency in infants with BPD perpetuates pulmonary vascular stress and remodeling, raising the risk for chronic PH [3]. In this cohort, preterm infants undergoing interventional PDA closure <2 kg were excluded.

In our study, invasive RHC provided critical data for evaluating the feasibility of shunt closure, especially in borderline cases. Lehner et al. and Salavitabar et al. advocate for the use of AVT and balloon occlusion to distinguish patients with reversible PH from those with fixed pulmonary vascular disease [5,9]. This approach allows for safe closure in infants with elevated PVR when a favourable response is documented. In our cohort, only 10 patients had an AVT, but the only 2 patients with negative response to AVT were deemed not feasible for direct shunt closure and were treated with PH medication before shunt closure (treat&repair approach). A “treat-and-repair” or “repair-and-treat” strategy was also described in some of the literature, involving staged closure and medical therapy. This approach was successful in select high-risk infants with borderline operability [10]. In all other cases without AVT, decision for shunt closure relied on a combination of clinical, echocardiographic and haemodynamic findings. Patient selection for shunt closure was mostly made prior to RHC, in fact only 6 of the 220 patients that were included were deemed not feasible for shunt closure. These findings echo previous research showing that successful closure without post-procedural PH deterioration is achievable when pre-intervention assessments confirm preserved left-to-right shunting and acceptable clinical status [5,9,10].

In our cohort, procedural success of interventional PDA closure was high, echoing findings by Shah et al. and Salavitabar et al., who reported over 95% device implantation success in PH patients [9,11]. This aligns with previous publications that showed low complication rates even in patients with elevated PA pressures [5,10,11]. Specifically, we had no periinterventional complications, but 28 patients were referred for surgical PDA closure. In this context it is worth mentioning, that we are dealing with an historical cohort and that practice has changed thanks to the experience with ELBW premature infants and new devices that are now available. Thanks to the experience with infants below 2 kg, interventionalist and referring cardiologists are know more keen on treating large ducts in small infants. In addition, new devices have become available. The Amplatzer Piccolo™ occluder, which is designed specifically for PDA closure in premature and low birth weight infants was not available throughout the time frame of the study. In large multicentre studies, the Piccolo has demonstrated clinical closure rates exceeding 99**%**, with a low incidence of serious adverse events [18,19]. These features make it the preferred choice in preterm neonates with large and hemodynamically significant ductus. Although originally developed for ventricular septal defect closure, the KONAR-MF™ occluder has been used successfully off-label for PDA closure in selected pediatric populations. The device features two symmetrical retention discs and a cone-shaped self-expanding waist, offering enhanced anchoring where procedural stability is essential. This structural advantage is particularly relevant in infants with elevated pulmonary pressures, where the risk of device embolization is heightened. Data from multicenter experiences confirm its efficacy and safety, with complete occlusion rates exceeding 90% and no major complications even in high-risk anatomy [20,21].

Several limitations to our study must be acknowledged. First, patients without a measured mean pulmonary artery pressure were automatically excluded from analysis. Additionally, infants with severe disease who were deemed too unstable to attempt PDA closure were excluded from this analysis since they never received an invasive assessment. This exclusion likely introduced a selection bias favouring more clinically stable patients and may underestimate the true burden or outcomes in the most critically ill subgroup. Moreover, the data are derived retrospectively, limiting the ability to establish causal relationships, especially since only few patients had PH at follow-up and predictors could not be statistically assessed. In addition, PVRi was available in only 39.1% of patients, and its calculation may be imprecise in PDA physiology because pulmonary arterial mixing can limit the accuracy of oximetry-derived pulmonary blood flow.

## 5 Conclusions

In this selected cohort of infants with PDA-associated pulmonary hypertension diagnosed before one year of age, persistent PH after transcatheter or surgical duct closure was uncommon. When present, it was observed mainly in infants with developmental lung disease or major comorbidities, rather than in those with isolated PDA. These findings support the feasibility of PDA closure in infants with PH when candidacy is guided by an integrated clinical, echocardiographic, and haemodynamic assessment, with careful attention to comorbidity status rather than pulmonary artery pressure values alone.

## CRediT authorship contribution statement

**Alessia Callegari:** Writing – review & editing, Writing – original draft, Investigation, Formal analysis, Data curation, Conceptualization. **Mathilde Meot:** Writing – review & editing, Data curation. **Damien Bonnet:** Writing – review & editing, Visualization, Supervision, Methodology, Investigation. **Sophie Malekzadeh-Milani:** Writing – review & editing, Visualization, Conceptualization.

## Ethics declaration

Written informed consent to take part in the study and to publish the article has been obtained from all participants or their legal representatives. The privacy rights of participants have been observed.

This study was performed in compliance with relevant laws, regulatory frameworks and guidelines where the research took place. This study was approved by the Necker Hospital. (Approval No. 20220729172242)

## Declaration of competing interest

The authors declare the following financial interests/personal relationships which may be considered as potential competing interests: Damien Bonnet reports a relationship with Janssen-Janssen, Novartis and MSD that includes: consulting or advisory. If there are other authors, they declare that they have no known competing financial interests or personal relationships that could have appeared to influence the work reported in this paper.
