# Supplementary material for: Pulmonary arterial hypertension before closure of a persistent arterial duct (PDA) in children <12 months: clinical implications and outcome
Source: Int J Cardiol Congenit Heart Dis. 2026 Oct 8;26:100718. doi: 10.1016/j.ijcchd.2026.100718 (PMC13717884; doi:10.1016/j.ijcchd.2026.100718)
Supplement: Supplementary file 1 [file mmc1.docx]

**Supplementary Table 1 (S1) – Characteristics of patients with persistent PH at 6 months**

| **ID** | **Age at cath (m)** | **Major comorbidity** | **mPAP (mmHg)** | **mPAP/mAo** | **Qp/Qs** | **PDA management** | **PH treatment at 6 months** | **Subsequent outcome** |
| --- | --- | --- | --- | --- | --- | --- | --- | --- |
| 1 | 2.9 | BPD, CHARGE syndrome | 27 | 0.49 | NR | PDA closure | O₂ | PH persisted; subsequently died from respiratory failure |
| 2 | 2.4 | LDD, diaphragmatic hernia | 29 | 0.63 | NR | PDA closure | O₂ | PH resolved |
| 3 | 2.5 | LDD | 50 | 0.85 | 0.69 | Delayed PDA closure | Sildenafil | PH resolved |
| 4 | 2.0 | LDD | 77 | 0.90 | 2.18 | Delayed PDA closure | Sildenafil | PH resolved |
| 5 | 0.7 | Alveolocapillary dysplasia | 37 | 1.00 | NR | No PDA closure | Bosentan + sildenafil | Persistent PH at 12 months and long-term follow-up |
| 6 | 8.7 | LDD, Trisomy 21 | 33 | 0.63 | 2.71 | Treat-and-repair with delayed PDA closure | Sildenafil | PH resolved by 12 months |
| 7 | 5.4 | Severe BPD | 30 | 0.55 | NR | Treat-and-repair; surgical PDA closure at 10 months | Sildenafil | Persistent PH at 12 months and long-term follow-up |
| 8 | 6.9 | BPD | 59 | 1.00 | 0.94 | No PDA closure | O₂ | PH persisted; subsequently died from respiratory failure |

Abbreviations: BPD, bronchopulmonary dysplasia; LDD, lung developmental disease; mAo, mean aortic pressure; mPAP, mean pulmonary artery pressure; NR, not reliably available; PH, pulmonary hypertension.
